# A Literature Survey with the Focus on Magnetically Coupled Wireless Power Transfer Systems Developed for Engineering and Biomedical Applications

**DOI:** 10.3390/mi14040786

**Published:** 2023-03-31

**Authors:** Lida Kouhalvandi, Serdar Ozoguz, Mohsen Koohestani

**Affiliations:** 1 Department of Electrical and Electronics Engineering, Dogus University, Istanbul 34775, Turkey; 2 Department of Electronics and Communication Engineering, Istanbul Technical University, Istanbul 34467, Turkey; 3 Department of Electrical and Electronic Engineering, ESEO School of Engineering, 49107 Angers, France; 4 Institute of Electronics and Telecommunications of Rennes, University of Rennes 1, 35042 Rennes, France

**Keywords:** wireless power transfer (WPT), magnetically coupled, engineering, biomedical applications

## Abstract

Wireless power transfer (WPT) is the transmission of electrical energy to other external/internal devices without the need for wire connection. Such a system is useful to power electrical devices as a promising technology for various emerging applications. The implementation of devices integrated with WPT alters the existing technologies and enhance the theoretical concept for future works. Over the last decade, various studies have been conducted on the applications of magnetically coupled WPT systems, where a general overview over such devices would be beneficial. Hence, this paper presents a comprehensive review over various WPT systems developed for commercially existing applications. The importance of WPT systems is first reported from the engineering point of view, followed by their uses in biomedical devices.

## 1. Introduction

The use of resonant magnetically coupled wireless power (WPT) systems is developing day by day to power various applications as diverse as radio frequency identification (RFID) tags, consumer electronics, healthcare, electric vehicle charging [1,2,3,4]. Inductive WPT technology is able to provide a low power level without depending on the physical connections making it interesting not only for engineering but also for implanted biomedical applications [5,6,7,8,9].

Generally, there is a compromise among operating range, achievable transfer efficiency and frequency bands of WPT systems [10]. At very low frequencies (e.g., 3 kHz), a very high efficiency (as high as 93%) can be achieved for very short distances (up to 6.5 cm), whereas at high frequencies (e.g., 13.56 MHz), an 40% efficiency is achievable for operating distances up to 2 m. At microwave frequencies (above 1 GHz), efficiencies higher than 50% is attainable for very long distances over several kilometers.

WPT systems can be used depending on the input impedance of the connected devices [11]. In [12], a methodology was presented to compensate the variation of load resistance. That approach is based on the active maximum efficiency point tracking method where an active single-phase rectifier, an auxiliary measurement coil with a corresponding control method were employed.

To effectively use WPT systems, the specifications of the target application are also of great importance. In [13], to obtain a suitable efficiency with an acceptable output voltage, single-switch regulated resonant wireless power receiver with hybrid modulation was designed. Yin et al. presented the single-switch step-up resonant inverter with a series–series compensated network to achieve the highest possible WPT efficiency [14], where the presented topology resulted in stable DC voltage. Another specification to be considered is the flexibility of the WPT systems for the use of customers. To tackle that problem, in [15], a controllable inductor was presented to extend the the soft switching region. Optimizing the area of the overall system is another important parameter. In [16], a methodology based on the Kirchhoff’s law and Maxwell’s mutual inductance formulations was proposed to reduce the overall area. The alignment between the transmitter (Tx) and receiver (Rx) elements has also to be considered [17]. In [18], a new method insensitive to misalignment is presented where in the Rx and Tx sections, instead of planar coils, two orthogonal coils were attached together leading to also cover longer ranges. The transfer distance variation is another concern in WPT systems specifically used in wireless charging applications. In [19], a planar Tx structure consisting of the multiple bidirectional sub-coils was developed to keep up an acceptable efficiency (>50%) at distances up to 70 mm. Beamforming concept was studied in [20] for the magnetic-based WPT systems where employing the multiple Tx and Rx coils provided a higher efficiency and reliability. For the small power levels, zero-voltage switching (ZVS) was presented in [21,22] to robust the power transmission at different distances.

This paper devotes to provide a summary of various circuits and technologies used for the design of WPT systems employed for emerging applications. For that purpose, the remaining of this manuscript is as follows. Section 2 presents the WPT systems from the engineering point of view whereas Section 3 summarizes the WPT systems that are designed for biomedical applications. Section 4 concludes this work and provides future perspectives.

## 2. Integrated and Magnetically Coupled WPT for Engineering Applications

As far as the engineering applications is concerned, WPT systems have been widely studied with the aim to enhance the output specifications such as transfer efficiency, operating range, and output power levels. The main outcome of the papers considered in the present survey is summarized in Table 1.

**Figure 1:**
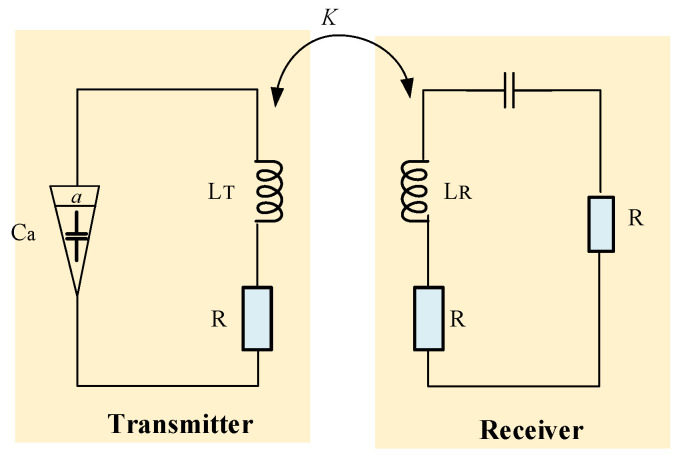
Schematic of the WPT system with fractional-order capacitor ($C_{a}$).

Typically, the WPT systems include integer-order components as inductors and capacitors. Jiang et al. presents fractional-order WPT that includes a fractional-order capacitor (Figure 1) based on the insensitivity of circuit on the resonant frequency where the two important transfer efficiency and output power parameters were stable and consistent [1].

**Figure 2:**
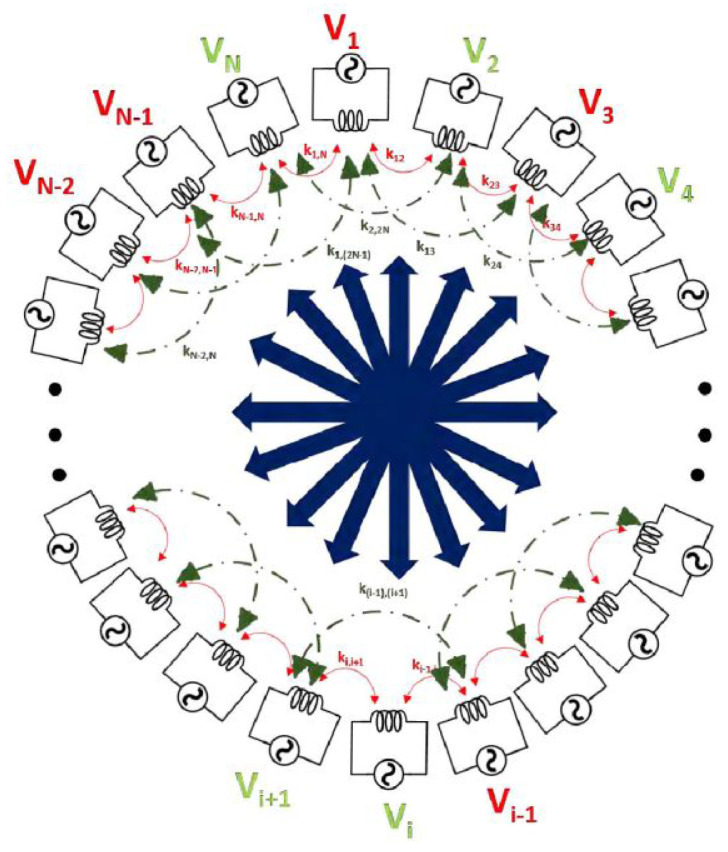
Multiple access WPT network presented in [23].

In [23], power flow selectivity and scalability specifications were investigated for a multiple access WPT. In that work, code division multiple access wireless power transfer (CDMA-WPT) was presented that let to achieving a stable power flow among various connected networks of devices. Figure 2 shows the multiple transceivers at the same time as well as the use of the CDMA-WPT system where the zero power factor was employed to achieve the orthogonal codes.

As previously mentioned, the performance of WPT systems depends highly on the load resistance. In [24] a matching network consists of lumped capacitor and inductor was employed for the load conversion within the optimal range. In that study, the optimal load was converted from 10 Ω to 600 Ω while the efficiency was remained as high as 89%.

**Figure 3:**
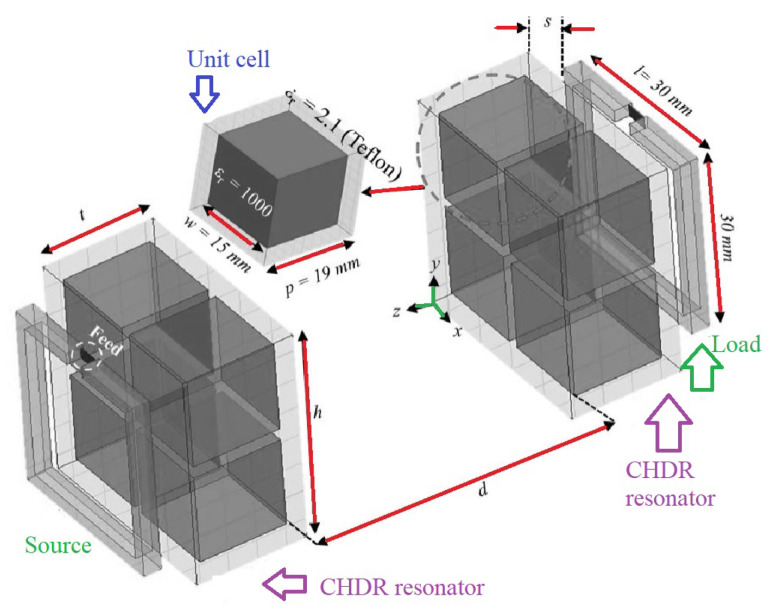
A WPT system with cubic high-dielectric resonator.

Das et al. presented a metamaterial-coupled WPT, shown in Figure 3, that is long-range and highly efficient based on the cubic high-dielectric resonator making the system less sensitive to the displacement of the receiver coil [25].

Another matter is the charging system of electric vehicles. In [26], a wireless charging system was presented, which uses a compensation topology for providing highly efficient overall system. That topology is based on the double-sided inductor-capacitor-capacitor (LCC) that was demonstrated to be suitable enough for WPT applications in electric vehicles.

**Figure 4:**
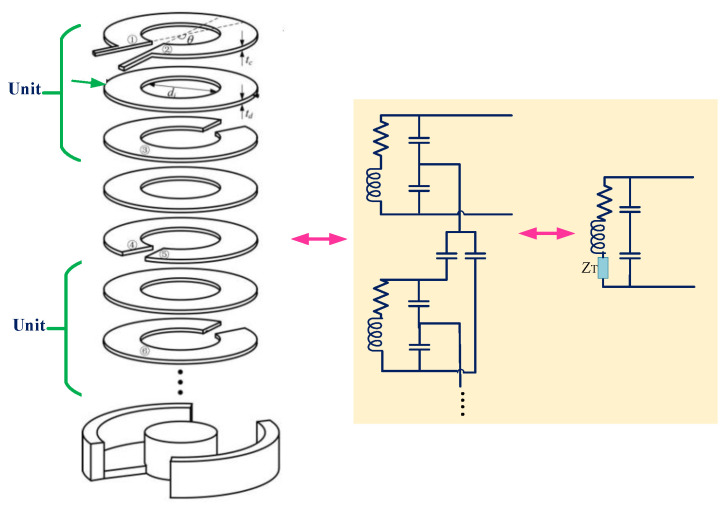
A WPT structure to enhance the Q-factor of the system.

As far as the efficiency of the WPT system is concerned, the quality factor (Q) of the inductive power transfer system is a vital parameter. To obtain a high-Q resonant structure, a new design on the resonant coil structure was proposed in [27]. Figure 4 depicts the design structure leading to increased Q-factor.

**Figure 5:**
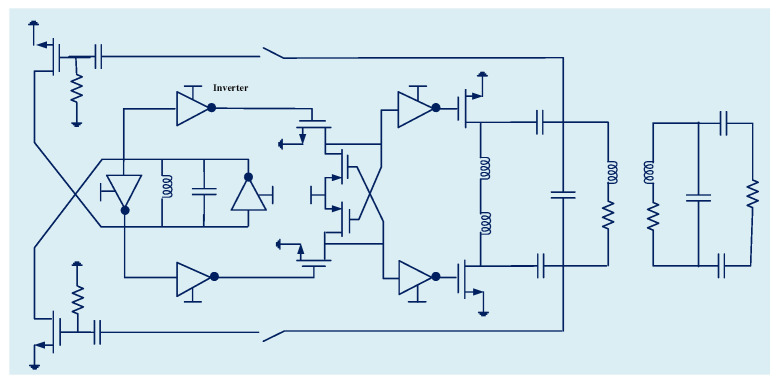
A WPT system with combined Tx design to achieve maximum power transfer.

**Figure 6:**
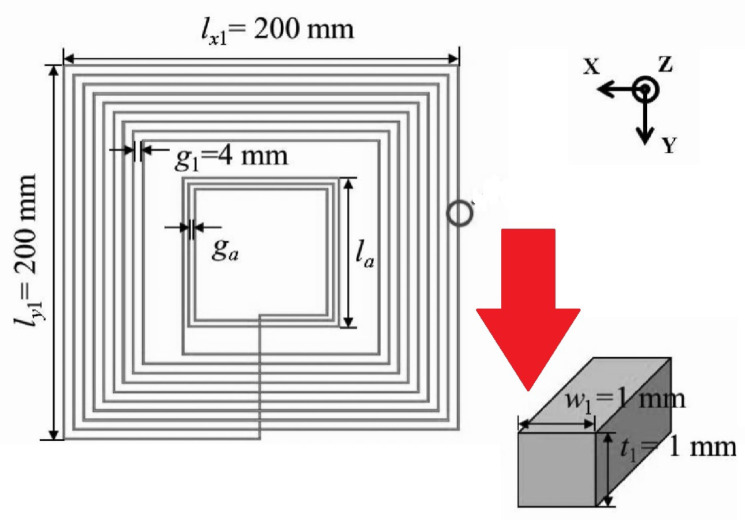
A WPT system with asymmetrical coil for increased coupling coefficient.

In [28], a closed-loop transmitter was designed to achieve a high power transfer efficiency. The designed Tx includes a source oscillator, power amplifier (PA), with its driver, matching network, and feedback circuitry (Figure 5). For highly efficient WPT systems, asymmetric coil structures were reported in [29]. Figure 6 shows the optimized coil structure including the three-turn spiral coil; details can be found in Table 1.

**Figure 7:**
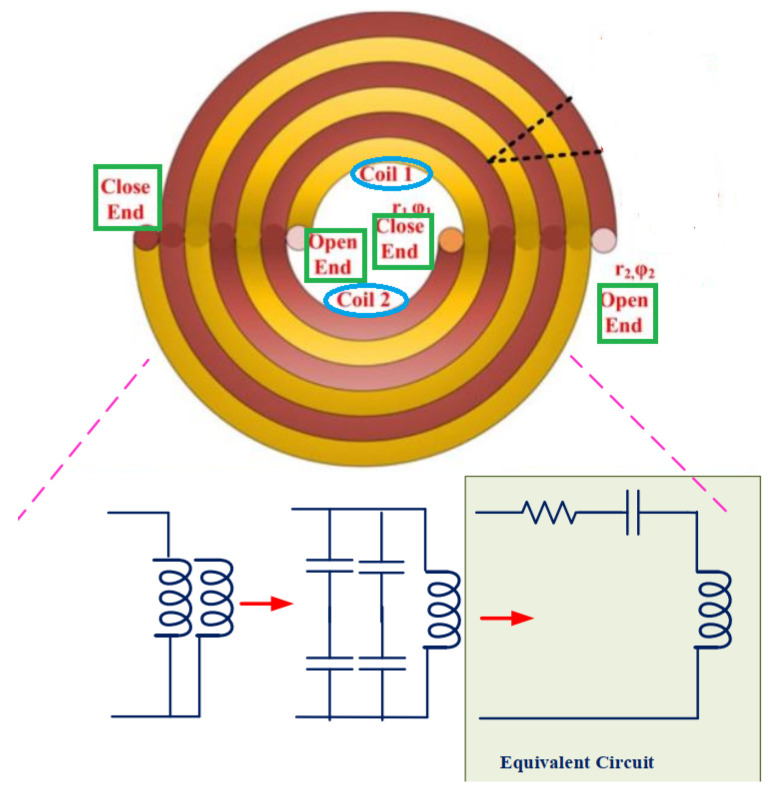
A WPT system with Bifilar coil with the equivalent circuit model.

In [30], a self-resonant Archimedean open bifilar spiral-coil system employed both in Tx and Rx sides was presented. In comparison with the conventional copper/Litz conductor, that topology was shown to provide a high power-transfer efficiency [30]. Figure 7 depicts the structure of the Bifilar coil together with its equivalent circuit model.

**Figure 8:**
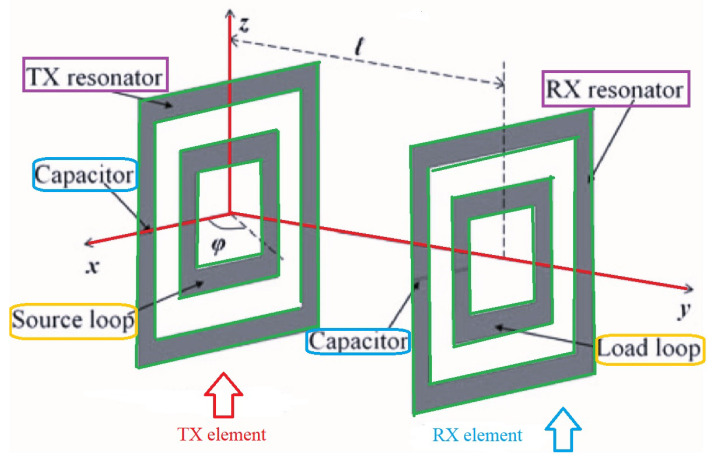
The configuration of the planar SCMR WPT system.

Liu et al. developed a planar strongly coupled magnetic resonance (SCMR) WPT system leading to keep the efficiency maximized in comparison with the inductive coupling [31]. The SCMR was demonstrated to be insensitive enough for the misalignment between the Tx and Rx elements. The typical structure of the SCMR is depicted in Figure 8.

**Figure 9:**
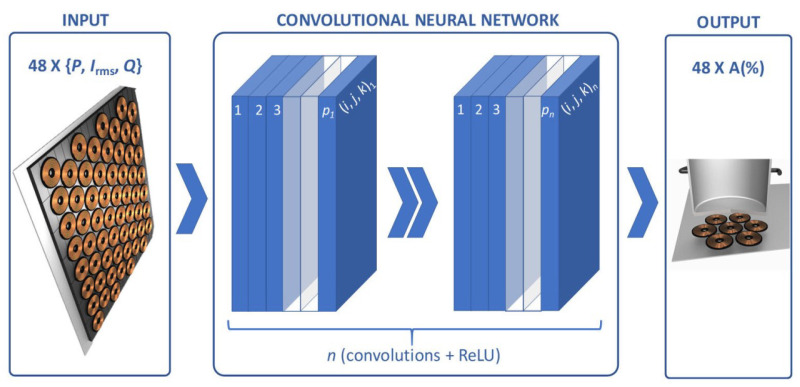
Demonstration of convolutional neural network presented in [32] where the input layer includes power (p), current ($I_{rms}$), and quality factor (Q).

Lucia et al. reported the magnetic coupling detection for heating applications where a deep learning approach was employed [32]. Figure 9 shows the use of convolutional neural network for estimating the area coverage of each inductor.

**Figure 10:**
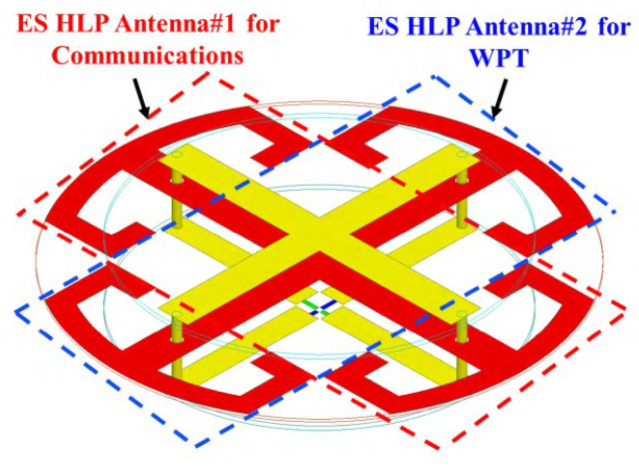
WPT system presented in [33] with Huygens linearly polarized antennas.

In [33], an electrically small Huygens dual-functional WPT system was presented where it operates in the 915-MHz band. The general structure is shown in Figure 10, which includes the Huygens linearly polarized (HLP) antennas. That system can easily find applications in Internet-of-Things (IoT) wireless communication systems.

**Figure 11:**
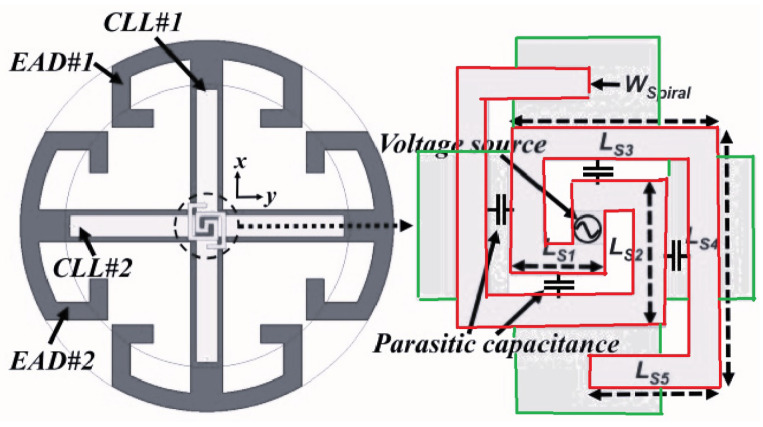
A WPT system with Huygens circularly polarized antenna, driven spiral line structure with capacitively loaded loop and Egyptian axe dipole.

In another study, small Huygens circularly polarized (HCP) rectenna (rectifying antenna) was presented in [34], where the rectifying circuit was integrated directly into the antenna. It also operates at 915 MHz and is highly capacitive. Figure 11 presents the general structure of the HCP antenna where the system includes an inductive input impedance. Additionally, a planar Huygens dipole rectenna was developed in [35] for WPT applications achieving a high gain (4.6 dBi).

**Figure 12:**
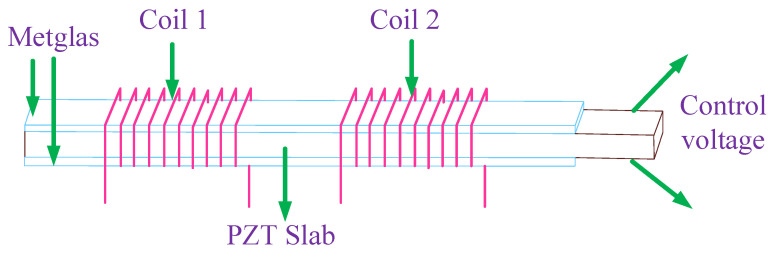
A WPT system with tunable mutual inductance device with lead zirconate titanate (PZT) slabs.

A dual-coil magnetic coupled resonance WPT system was reported in [36] with various tunability for the low- and high-frequency range. Figure 12 depicts the power transmitting test setup where magnetoelectric (ME) is composed of zirconate titanate (PZT) materials inserted inside two solenoid-type coils.

**Figure 13:**
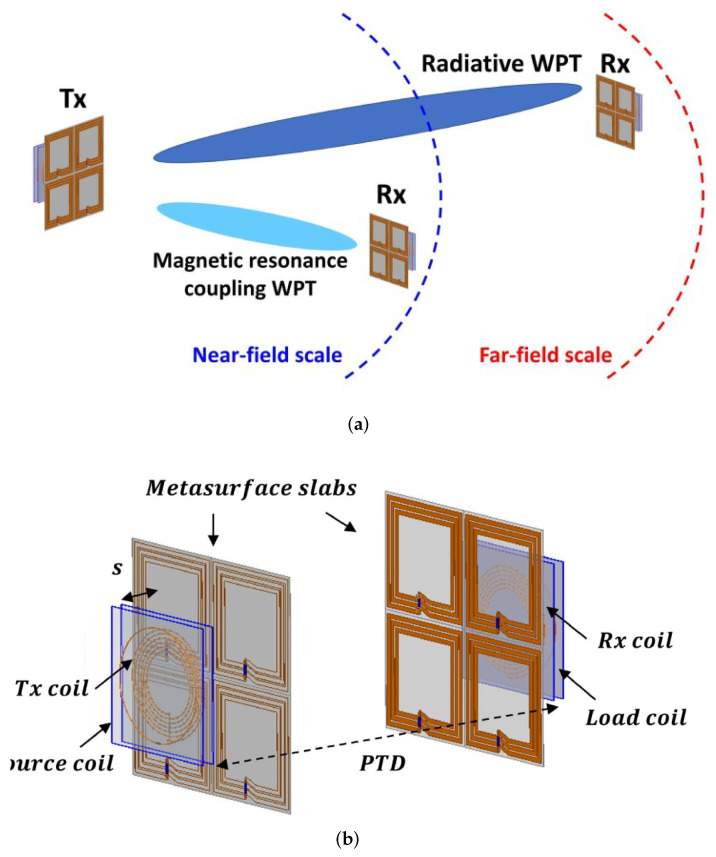
(**a**) Concept and (**b**) structure of the metasurface-based WPT presented in [37].

Lee et al. presented a metasurface-based multi-scale WPT that can work at 6.78 MHz in the near-field and at 433 MHz in the far-field regions [37]. Figure 13a depicts the concept of the developed metasurface WPT that is operating both at near- and far-fields whereas its structure is shown in Figure 13b. Compared to a no-slab case, the achieved power transfer efficiency was increased up to 50.1% with the metasurface slabs.

**Figure 14:**
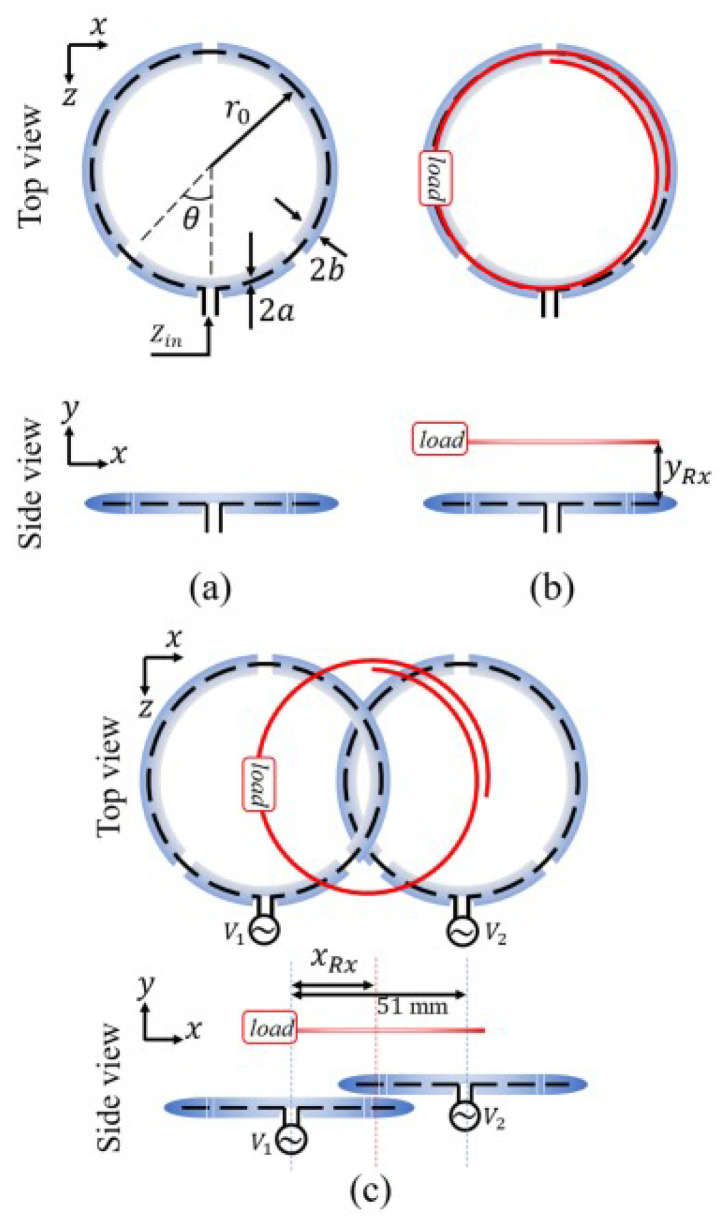
The WPT system configuration in [38]: (**a**) Tx coil in free space, (**b**) Rx in presence of the proposed Tx, (**c**) WPT system configurations with the Tx and Rx coils.

In [38], a WPT system based on two high-impedance coil (HIC) was developed. The Rx coil was positioned freely over the array of the Tx coils leading to a highly efficient performance. The Tx coils were designed in a way that the input impedance was high enough compared to the case where the Rx coil was placed near the Tx one. Figure 14 shows the proposed configuration leading to have an averaged efficiency of 93%.

**Figure 15:**
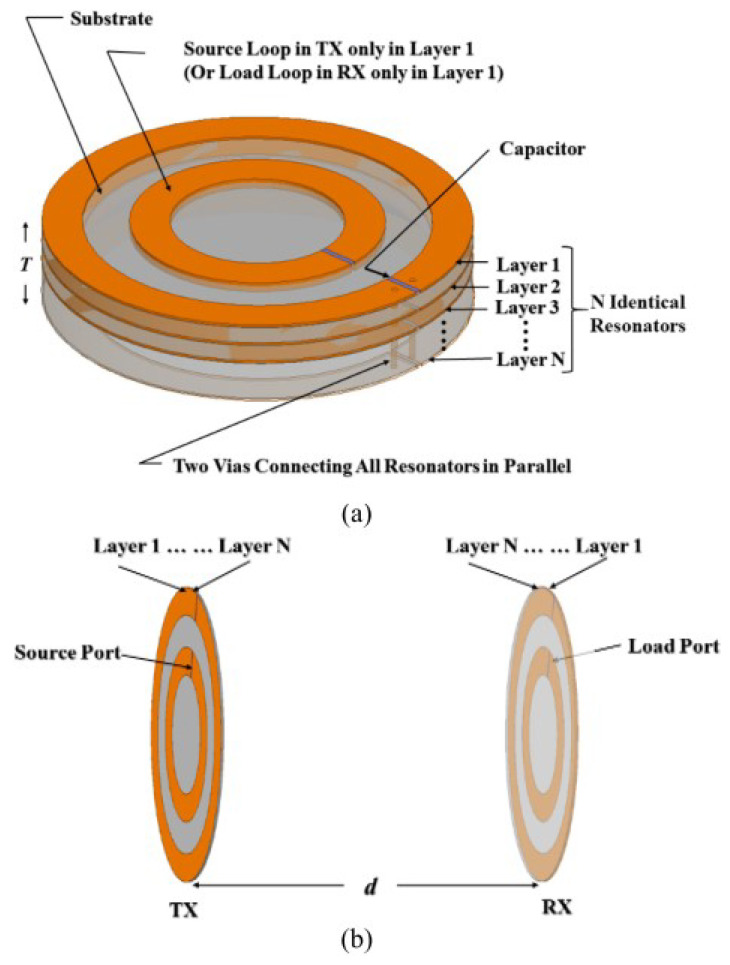
The planar multilayer elements SCMR WPT system developed in [16]; (**a**) geometry of the Tx and Rx elements, (**b**) general configuration of multilayer system.

Last but not the leastm with the aim to reduce the overall size of the WPT system, Bao et al. presented a planar multilayer elements SCMR WPT system [16]; its general structure is shown in Figure 15. Their results show that for the targeted efficiency is obtained with a 50% area reduction.

**Table 1:** A summary of the specifications of the magnetically coupled WPT systems studied in Section 2.

Ref.	Scope	Contribution	Specifications
[1]	Providing fractional-order WPT System.	Transfer efficiency with output power are insensitive to the resonant frequency.	Meeting the requirements of efficiency with output power only change within 1% when the receiver resonant frequency is reduced by ∓5%.
[23]	Enabling WPT among multiple Tx and Rx simultaneously.	Presenting code division multiple access wireless power transfer (CDMA-WPT).	Achiving 5 W output power with about 75% efficiency.
[24]	Presenting hybrid load matching method for WPT system.	Achieving high efficiency specification.	89% efficiency from 10 Ω (527.8 W) to 600 Ω (8.64 W)
[25]	Presenting a metamaterial-coupled WPT system.	Consisting of two cubic high-dielectric resonators.	More than 80% efficiency at short distances.
[26]	Integrating the compensated coil into the main coil structure.	Presenting a compact model results in reduced size.	Transferring 3.0 kW with 95.5% efficiency at an air gap of 150 mm.
[28]	Keeping high power transfer efficiency in the over-coupled region.	Presenting a closed-loop transmitter for wireless power transfer.	60% efficiency at highly over-coupled spacings around 10 mm.
[29]	Presenting Tx- and Rx-coil for magnetic resonant WPT systems.	Presenting high efficiency at medium distance.	96% efficiency at 50 mm and 39% efficiency at 300 mm.
[30]	Employing high temperature superconducting (HTS) wires in a WPT systems.	Enhancing the PTE in comparison with the conventional copper/Litz conductor.	PTE of 49.8% with the resonant frequency of 25 kHz.
[31]	Introducing strongly coupled magnetic resonance design.	Enhancing efficiency at larger distances.	40% efficiency for the entire 360° misalignment.
[32]	Providing highly flexible cooking surfaces.	Composing of multi-coil structures that is based on deep-learning approach.	Estimating the magnetic coupling between the coil and the induction heating load.
[33]	Presenting small Huygens dual-functional WPT systems.	Combining a Huygens linearly polarized (HLP) antenna and a highly efficient HLP rectenna.	Peak gain of 2.7 dBi in the 915-MHz industrial, scientific, and medical radio band (ISM band).
[34]	Presenting small Huygens circularly polarized (HCP) rectenna.	Providing a near-field resonant parasitic where the rectifier circuit is highly capacitive.	Efficiency of 90.6% in the 915 MHz ISM band.
[35]	Presenting single-substrate Huygens dipole rectenna.	Consisting of two metamaterial inspired near-field resonant parasitic (NFRP) elements.	Efficiency of 88% with a gain of 4.6 dBi.
[36]	Presenting dual-coil magnetic coupled resonance WPT system.	Presenting an energy transfer efficiency in overcoupled state.	Tunability of 56.5% in the low-frequency range and also a tunability of 16.6% in the high-frequency range.
[37]	Presenting metasurface-based multi-scale WPT system.	Working in both near-field scale and far-field scale.	Power transfer efficiency of 50.1 % at 433 MHz ISM band.
[38]	Presenting WPT system based on the two high-impedance coil—cable loop antennas.	Presenting a high the input impedance for the Tx.	High-frequency range (around 280 MHz) with efficiency of 93%.

## 3. Magnetically Coupled WPT Systems for Biomedical Applications

WPT systems provide a reliable solution regarding the power supply of biomedical devices [39]. This section deals with the recent advances of WPT systems developed for biomedical applications. The main messages of the papers considered in this survey is summarized in Table 2.

Since WPT systems operate with relatively high powers and produce strong electric and magnetic fields, they rise issues related to the compatibility with surrounding electronic devices and to the exposure safety. Hence, accurate assessment of user exposure is of great importance for WPT systems in body-centric applications. For the sake of completeness, a few research works are first reported analyzing local exposure induced inside the human body by a generic resonant WPT system.

**Figure 16:**
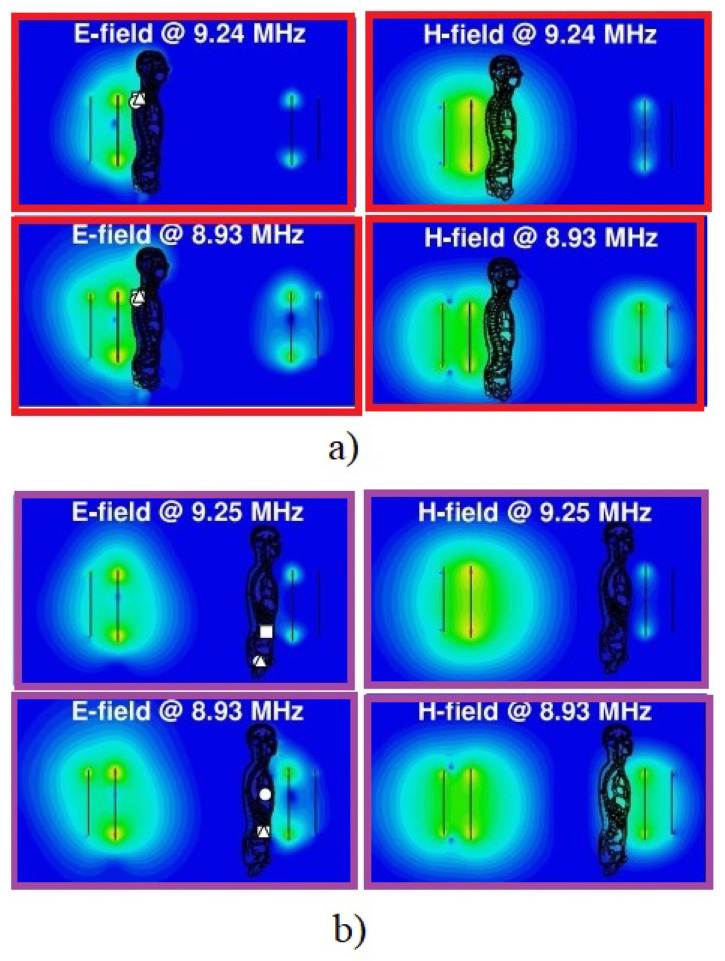
Electric and magnetic field distributions around the human body in proximity to the WPT system for an input power of 1 W; the body is more exposed to the generated fields by the (**a**) Tx and (**b**) Rx coil. Scale: in dB with 10 subdivisions ranging from −80 to 0 dBV/m and −60 to 0 dBA/m for *E* and *H*, respectively.

In [10], a methodology for the design of 10 MHz planar magnetically coupled resonant WPT systems was introduced with aim to design a a well-matched system with a maximized power-transfer efficiency for mid-range applications. A detailed dosimetric study was performed using a detailed high-resolution anatomical human body voxel model to evaluate the exposure levels with respect to the ICNIRP basic restrictions considering $E_{99}$, $J_{1cm^{2}}$, and specific absorption rate (SAR) (local and whole body averaged SAR) as exposure metrics. Figure 16 depicts the distributions of the electric and magnetic fields inside the body for different locations of the body between the Tx and Rx coils.

**Figure 17:**
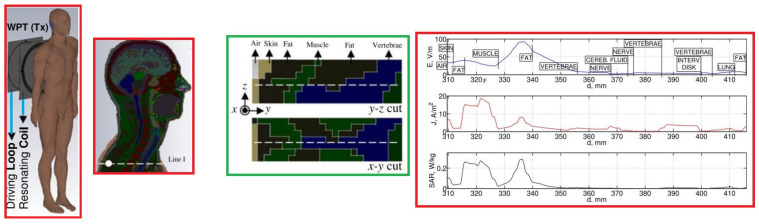
The exposure scenario with Tx part of a WPT system in proximity to the Duke human body model showing the distribution of E, J, and SAR inside the model along line I.

In [40], the local exposure induced inside the human body by a generic resonant WPT system operating at 10 MHz was analyzed in detail demonstrating the impact of electromagnetic properties of biological tissue, as well as of the body geometry and size on local distribution of the EM field inside the body. Figure 17 shows the distribution of the studied dosimetric quantities (i.e., E, J, and SAR) for a considered exposure scenario.

**Figure 18:**
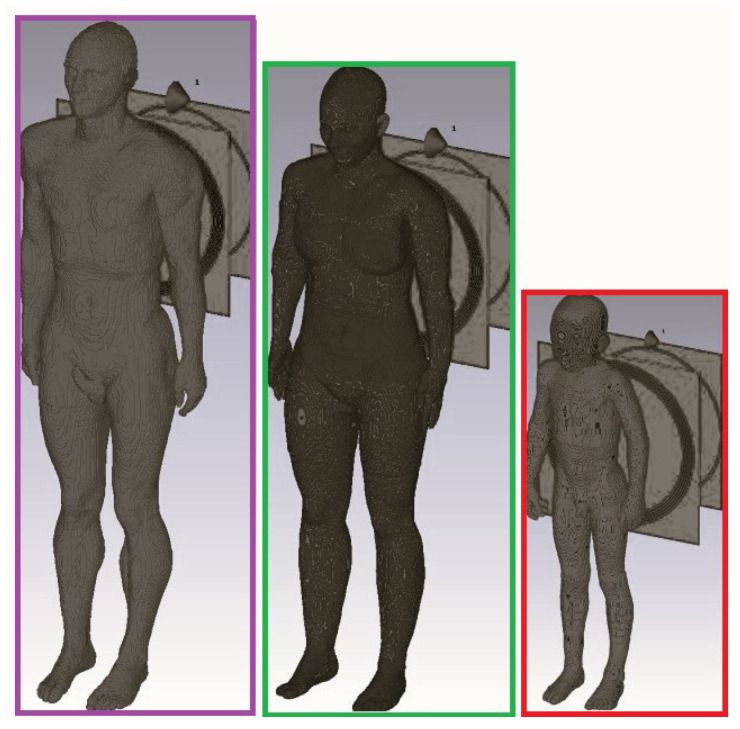
Tx part of a resonant WPT system in proximity to Duke, Ella, and Thelonious virtual family body models.

In [41], a dosimetric study was conducted with the aim to compare the exposure levels among children and adults when exposed to a 10 MHz resonant WPT (Figure 18). The exposure levels with respect to the ICNIRP basic restrictions were evaluated in terms of dosimetric quantities ($E_{99}$, $J_{1cm^{2}}$, and local and whole body SAR).

**Figure 19:**
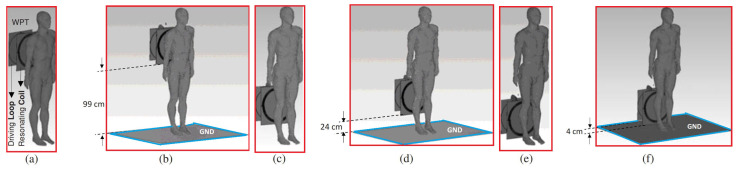
Tx part of a 10 MHz WPT system in proximity to a grounded and ungrounded Duke model at different locations with respect to the ground: (**a**,**c**,**e**) ungrounded, (**b**,**d**,**f**) grounded.

In [42], with the aim to assess the importance of the WPT system location with respect to the ground plane, the impact of the latter on the exposure dosimetric quantities was showcased considering a grounded and ungrounded Duke body model exposed to a resonant WPT system for different distances between the Tx part of the system and the ground (Figure 19).

**Figure 20:**
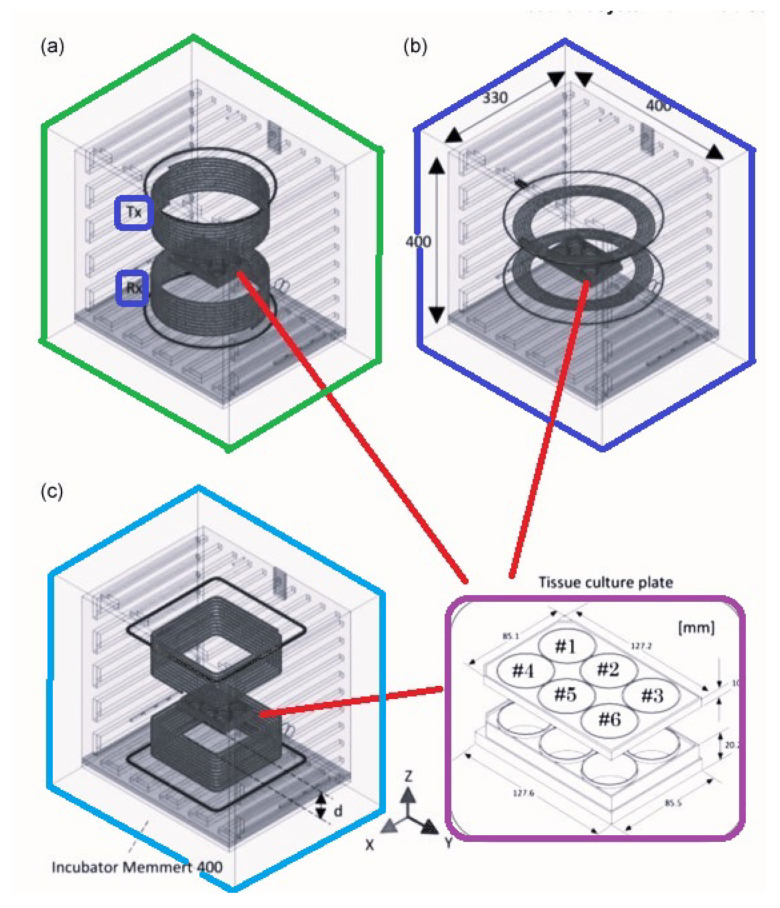
WPT exposure systems of different loops/coils geometries optimized according to the methodology for a 10 cm distance between Tx and Rx coils providing a maximized transfer efficiency greater than 90% at the frequency of interest: (**a**) cylindrical with circular cross-section, (**b**) annular, (**c**) cylindrical with square cross-section.

In [43], an exposure system for in vitro studies was designed to emulate the exposure of a monolayer of cells to a 13.56 MHz WPT system, aiming at maximizing the SAR uniformity on the plane where the layer is cultured, as well as SAR efficiency (defined as SAR over the input power) within the size constraints of a standard incubator (Figure 20). The concluding contributions of that study are summarized in Table 2.

**Figure 21:**
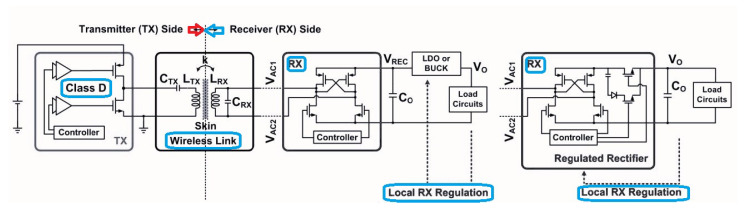
The overall presented Tx and Rx structures based on the CMOS technology.

In [44], a technique, namely as constant-idle-time control, was introduced for designing Tx and Rx chips of the developed system (Figure 21). In the designed chip, no wires and no discrete components were used leading to reduce the its complexity. The whole circuit was designed with the 65 nm CMOS technology providing a maximum output power of 49.4 mW at 13.56 MHz frequency.

Based on the method presented in [2], namely as line-array technique, miniaturized medical devices can be designed. In that study, a line-array Tx structure was considered leading to enhancing the power gain where the overall structure includes lines instead of coils. The operational frequency band is at Ku-band (i.e., 12–18 GHz) and the presented configuration is fabricated in 65 nm CMOS technology.

In [45], the design of a WPT was reported where it does not require a dc–dc converter for controlling the output power. The structure results in a fast transient response since it is insensitive to the coupling and load variation and can be used in retinal prosthesis applications.

A triple-loop WPT system was presented in [46] that can be used for implantable biomedical devices. That proposed system involves three sections as closed-loop global power control, adaptive Tx resonance compensation, and automatic Rx resonance tuning. For activating these three loops, a sequential control algorithm was used to provide a reliable stability.

WPT-based deeply implanted biomedical devices help minimize the patient exposure to tissue heating. In [47], parallel insulated capacitive electrodes were employed where the system was validated through the circuit coupled finite-element analysis for the fast determination of the output power.

In [48], an effective WPT through three-coil inductive link was presented where the poor received power stability (RPS) was investigated in terms of position and orientation stability. The overall achieved PRS was found to be around 68.7% where the transferred power is at least 570 mW. The presented design led to improve the power transfer efficiency.

**Figure 22:**
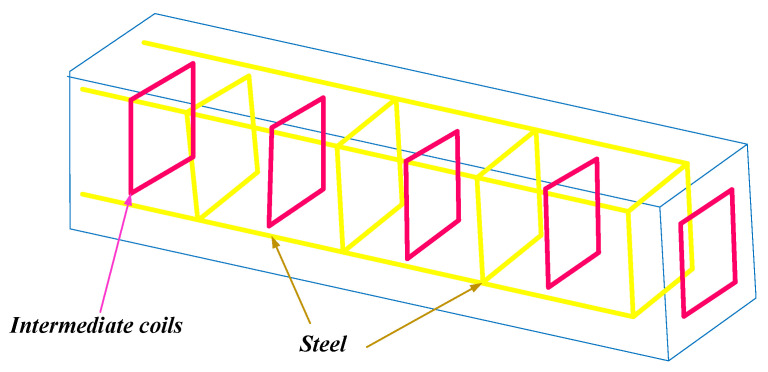
The overall structure of the inductively coupled WPT system.

An inductively coupled WPT was employed in [49] to power sensor nodes providing an overall system transmission efficiency of 47.7% for a 51.5 cm distance with 0.6 W input power. Figure 22 presents the overall model of inductive power transfer system.

In WPT systems, mutual coupling between coils is usually used to optimize the overall performance. In [50], for decreasing the constraints on the coil layouts, mutual coupling, mutual capacitance and relative polarity were investigated demonstrating a 40% efficiency performance for a 50 mm distance at 5 MHz.

**Figure 23:**
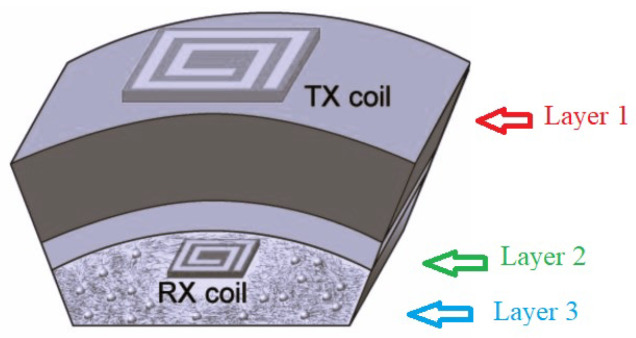
Exposure scenario with the implanted Tx and Rx coils.

In implantable medical devices, planar coils are mostly used due to their safe performances. In [51], planar square-spiral coils, shown in Figure 23 were designed through automated design method. Based on the design constrains, the optimal coil pair for the maximum efficiency was demonstrated to be achievable.

**Figure 24:**
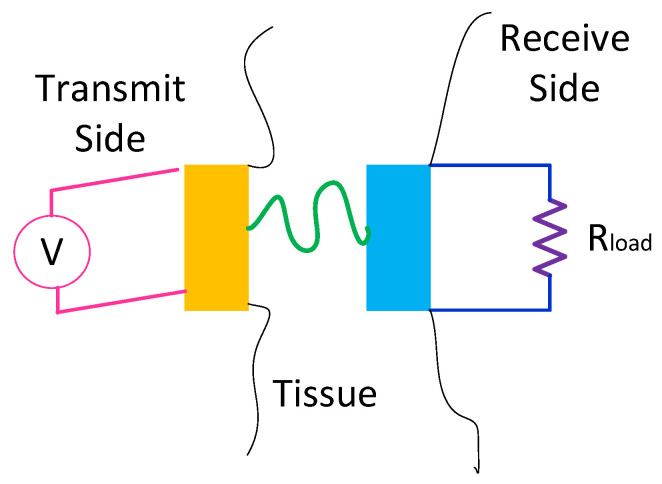
The UTET structure.

In wireless communication systems employed for biomedical applications, ultra-wideband technology is also interesting due to low power spectral density [52,53,54,55]. Another example of WPT technology for medical uses is towards the ultrasonic power transfer through piezoelectric devices. A low-power and non-invasive practical ultrasonic transcutaneous energy transfer (UTET) was presented in [51] where the Tx section is connected into the voltage source whereas the Rx is connected into the electrical load (Figure 24). The passive capacitive parametric ultrasonic transducer was designed in [56] that can be used for medical implants, and sensor networks. That design does not require a DC bias or a permanent charge in which a 1-D lumped parameter model was used.

**Figure 25:**
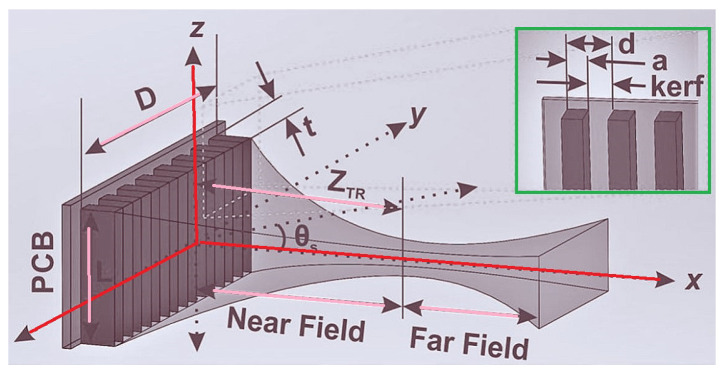
A linear phased array with a conceptual beam shape.

Another study considered the Rx implanted in their proposed WPT system [57]. Figure 25 shows the overall schematic of the phased array design with a conceptual beam shape, including near- and far-field zones, which can be operated at 1.1 MHz with the overall output power and transfer efficiency of around 6 mW and 0.14%, respectively.

**Figure 26:**
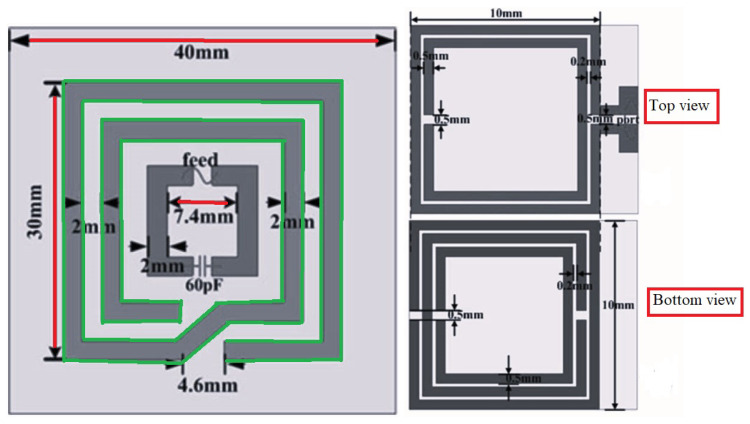
A WPT system integrated with the metasurface: Tx (left) and Rx (right) coils.

In the high frequency band (e.g., 430 MHz), a WPT system integrated with the metasurface was presented in [58] as shown in Figure 26. The presented design was simulated inside the body demonstrating an overall $S_{21}$ level of −27.9 dB for distances up to 30 mm.

**Figure 27:**
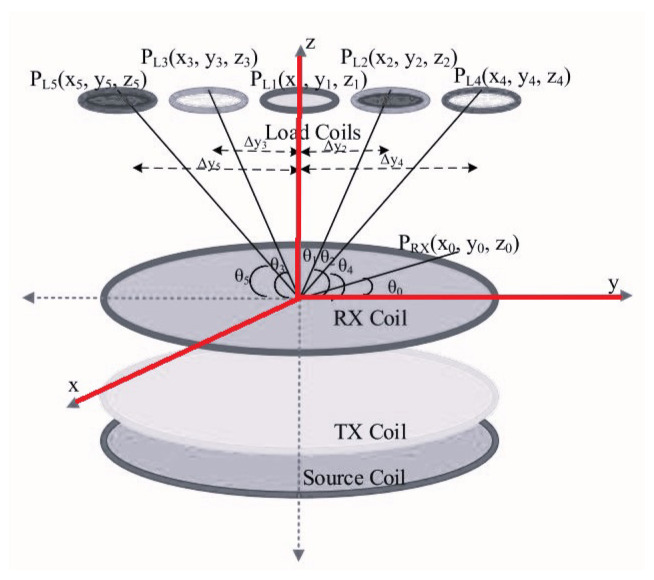
The configuration of the WPT system developed in [59].

In [59], in order to provide simultaneous independent power transfer, three coils in the transmitter side was used with the configuration seen in Figure 27. The multiple load coils system is evolved by considering the induced load current and the load coil position.

**Figure 28:**
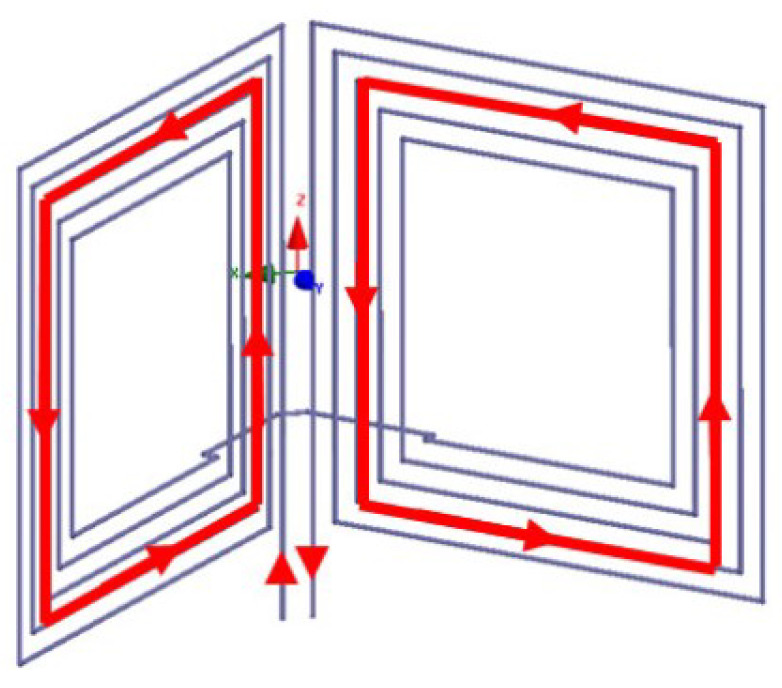
The WPT system proposed in [39] for implanted medical devices.

In [39], butterfly-shaped Tx was introduced leading to reinforce the power transfer efficiency of devices that can be used for implanted biomedical devices (Figure 28). This coil was supplied through the single power source obtaining maximum efficiency.

**Figure 29:**
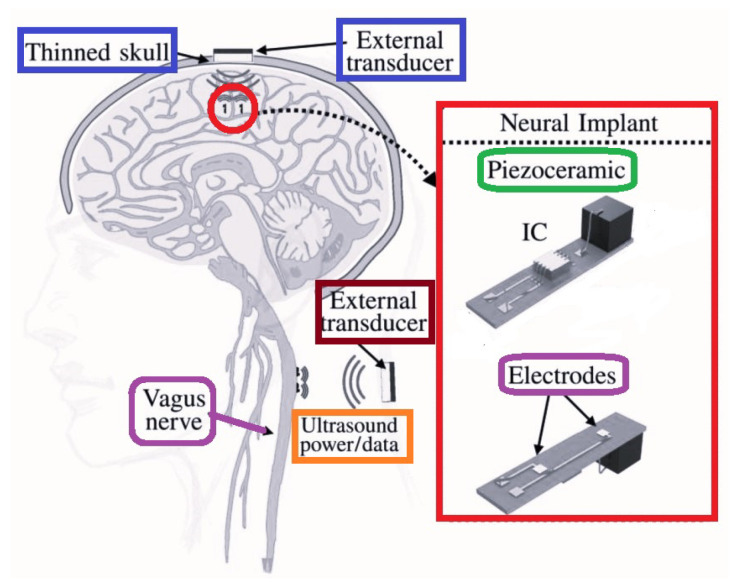
Acoustic power transfer for neural recording.

The acoustic power transfer, that is the free-floating implant for neural recording, was presented in Figure 29. That neural implant includes piezoelectric receiver, sub-mm IC and recording electrodes where the total area is only 2.7 ${\mathrm{mm}}^{3}$. The effectiveness of this device was verified through implementation in rats.

Neural implantable sensors are sensitive devices since a methodology for reducing the electromagnetic interference is required [55]. In [60], resonant capacitive-coupling was employed for that reason. The investigated methodology led to provide suitable healthcare. More details can be found in Table 2.

**Figure 30:**
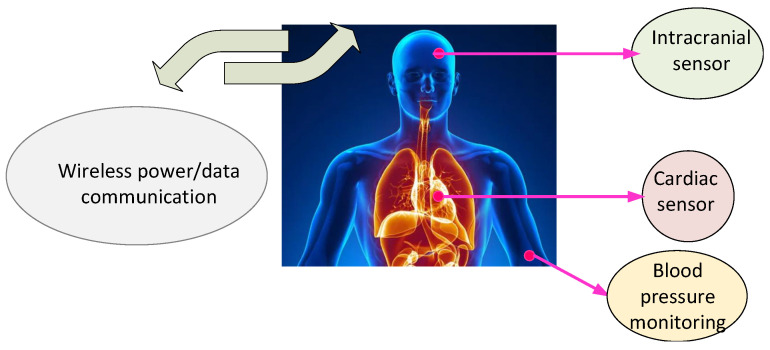
General view of wireless implantable sensors.

Figure 30 depicts the importance of wireless biosensors in medical treatments to increase the life quality [61]. The presented system was shown to be efficient for wireless powering of low-power medical devices in the industrial, scientific, and medical (ISM) frequency band.

**Figure 31:**
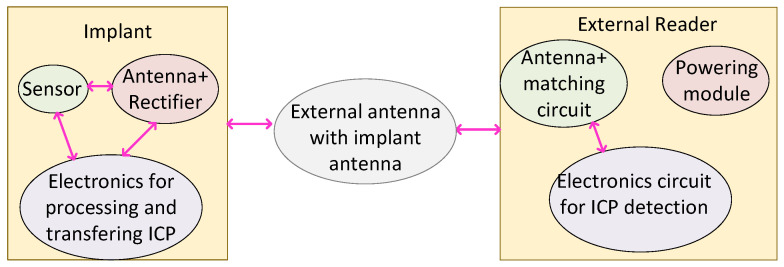
Remotely powered piezoresistive pressure Sensor for monitoring health issues.

For long-term monitoring of implanted pressure sensor, activating the piezoresistive pressure sensor is usually used. Figure 31 shows the general overview around the installed sensor leading to monitor the health problems [62].

In [63], the WPT system with RF transmission were developed for the use in implantable medical applications. The base implementation of the system was the spiral coil for magnetic resonant coupling achieving a 115 mW output power in the receiving side. The combination of WPT system with antenna achieve a power transfer efficiency of 47.2%.

**Figure 32:**
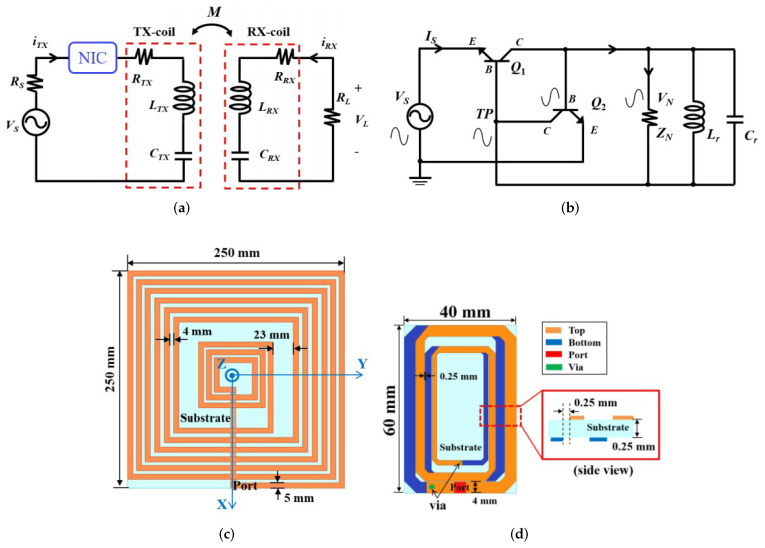
(**a**) proposed WPT system with the use of NIC, (**b**) the circuit-level of NIC, (**c**) TX-coil, (**d**) Rx-coil presented in [64].

In order to enhance the efficiency of the WPT system for medical uses, a negative impedance converter (NIC) based on the non-foster theory was presented in [64]. The presented converter consists of the transistor, voltage divider, capacitor, and inductor achieving a 96% efficiency. Figure 32 shows the developed WPT system as well as the open circuit of NIC, Tx- and Rx-coils configurations are presented.

In [65], an implantable rectenna was presented for power transferring, which comprise a compact planar inverted F-antenna. That structure is operating at 401–406 MHz with an overall size is 16 × 14 × 1.27 ${\mathrm{mm}}^{3}$. In [66], a 2.4 GHz rectifying patch antenna was designed to implement the principles of wireless power transfer through radiating antennas. That design operates up to 15 cm where the maximum loss is 7.5 dB and the efficiency is 40% at 0 dBm. For farfield WPT systems, a wireless power link is presented in [67], where a circularly polarized implantable antenna was designed. That structure operates from 889 to 924 MHz with peak gain of −29.2 dB. A compact rectenna for WPT system was presented in [68] operating at 868-MHz/915-MHz, where the dc power is 30 μW with 35% efficiency. In [69], an implantable rectenna based on WPT system was developed that operates at 902.8–928 MHz where rectifying efficiency was 24% is obtained for a 10 KΩ load.

**Table 2:** Take-home messages of the WPT systems studied for biomedical applications presented in Section 3.

Ref.	Scope	Contribution	Specifications
[2]	Presenting Tx array structure where lines are used instead of coils.	Keeping the line arrays antimisalignment.	Operating at Ku-band with CMOS Rx rectenna and a printed circuit board (PCB) Tx line array. The size of system is 100 µm × 100 µm where the gain is improved by 17.3 dB at a power transfer range of 2.5 mm.
[10]	Presenting a methodology for the design of printed magnetically coupled resonant considering human safety regulations.	Introducing a well-matched system with maximized power-transfer efficiency without the need for additional matching circuits connected to the system.	Working at 10 MHz with the input power at the range of 22–675 W.
[39]	Presenting a butterfly-shaped transmitting coil.	Enhancing the efficiency of the system in the distance, angle, and axial misalignment tolerances.	Presenting low specific absorption rate that is safe for medical applications.
[40]	Considering local exposure induced inside the human body at locations where the magnetic field polarization is either parallel or perpendicular.	At Presented locations where the H-field polarization is perpendicular to the body, the maximum E appears in deeper tissues compared to the locations having a parallel polarization.	Working at 1 MHz demonstrating that the non-uniform distribution and location of maximum of the E-field inside the body can be locally interpreted based on the tissues intrinsic impedance contrast.
[41]	Assessing the exposure due to a representative WPT system in three different human body models, i.e., adult male and female as well as a child	Demonstrating that the exposure to a child is the same or lower than in an adult.	Body dimensions play an important role being more pronounced for peak RMS values of $E_{99}$ and $J_{1cm}^{2}$ compared to those obtained for ${\mathrm{SAR}}_{10g}$ and ${\mathrm{SAR}}_{wb}$.
[42]	Comparing the exposure of a grounded and ungrounded human body.	Presenting independently of the WPT system distance where the ground, peak dosimetric values are much higher in grounded than ungrounded scenarios.	$SAR_{10g}$ is the most restrictive dosimetric quantity.
[43]	Presenting the design of a resonant system for in vitro studies.	Equipping with cylindrical coils and square cross-sections led to a high EM field uniformity in the in vitro biological samples.	Operational frequency of 13.56 MHz demonstrating that the uniformities in E and SAR were limited among the wells to a maximum of 7.9% and 5.5%, respectively.
[44]	Presenting a complete WPT system with Tx and Rx chips.	Achieving output voltage regulations by the proposed constant-idle-time control.	17.5% efficiency improvement where the chip is fabricated with 65-nm CMOS technology.
[45]	Presenting a resonant power converter.	Presenting an enhanced efficiency with minimum sensitivity.	Keeping soft switching against large variations in the loads.
[46]	Presenting a triple-loop WPT system.	Presenting a design where it opposes coupling and load variations and also compensates for changes in the environment surrounding the inductive link.	10.5% efficiency at 13.56 MHz.
[48]	Presenting a magnetic resonant-based WPT system.	Providing enhanced efficiency with stable power.	Power efficiency of 79.2%.
[49]	Presenting a multicoil inductive power repeater system.	Performing coil as a power relay and also supplies energy.	Efficiency of 47.7% at a long distance of 51.5 cm.
[51]	Presenting a methdology for automated design of planar square-spiral coils.	Generating the idealized design parameters for enhancing power transfer efficiency.	Reducing in design time where all the design process can be done in few minutes and it is automated.
[55]	Presenting a theory for near-field resonant inductively coupled WPT.	Developing ultrasonic, mid-field, and far-field coupled WPT technologies.	Proving the efficiency of the presented method for the coupled WPT systems.
[56]	Presenting a 1-D lumped parameter model for passive capacitive parametric ultrasonic transducer.	Proving that the presented design does not need a DC bias or a permanent charge.	Presenting highly efficient power transfer.
[57]	Presenting the theory and design methodology of ultrasound WPT system.	Presenting iterative design procedure to enhance the power transfer efficiency.	6 mW power with the power transfer efficiency of 0.14%.
[58]	Presenting implantable magnetic coupling resonate WPT system.	Employing conformal strongly coupled magnetic resonator coil for constructing power link.	15.7 dB coupling enhancement.
[59]	Presenting an approach for simultaneous independent wireless power transferring.	Employing three coils at the Tx side.	Performing on the five loads, power transfer and force generation at frequency splitting.
[47]	Presenting a capacitively coupled conductive power transfer method.	Providing safe transfer of power into the body.	Running at 6.78 MHz, delivering 10 mW deep into the body.
[60]	Presenting a capacitive-coupled power transfer method.	Developing a resonant capacitive-coupling method for WPT system.	Efficiency more than 24%.
[62]	Presenting the results of pressure measurements after using impalnted sensor.	Presenting sensor activation by using inductive power transmission.	Resulting in power of 4.47 mW.
[63]	Presenting ultracompact design of biomedical implantable devices.	Designing integrated WPT with radio frequency transmissions.	Gain of −15.71 dBi with power of 115 mW.
[64]	Presenting a negative impedance converter.	Increasing the system efficiency that is based on the non-foster theory.	Introducing Efficiency more than 30% for a distance more than 100 mm.
[65]	Presenting an arm-implantable rectenna.	Supporting a planar inverted F-antenna and a rectifier.	ISM frequency band
[66]	Presenting a radiating near-field method.	Employing the principles of wireless power transfer using radiating antennas.	Performing up to 15 cm, showing a maximum loss of 7.5 dB.
[67]	Studying a wireless power link with circular polarization.	Employing the the system for far-field wireless power transmission.	915 MHz frequency with input power of 25 dBm and peak gain of 8 dB.
[68]	Characterizing a compact rectennas for wireless power transmission application.	Employing rectennas for supplying power to a dcto-dc boost converter.	868-MHz/915-MHz frequency band with power consumption of 9.45 mW and a dc voltage of 3 V.
[69]	Presenting a complete RF to DC wireless power transmission.	Employing implantable rectenna system.	902.8–928 MHz frequency band with input power of −20 dBm.

## 4. Conclusions

An overview of the use of magnetically coupled WPT systems developed over the recent years for various applications was presented. The systems were classified into engineering and biomedical categories. Such a study would give readers an overall vision not only on the current status of the WPT technology but also provides a quick access to the specifications (such as operational frequency and distance, transfer efficiency and power handling) of the developed WPT systems. As perspectives, intelligent-based methods have to be developed for the design of WPT syetems to make them for instance insensitive to misalignment. Additionally, the interest of such systems for powering devices developed for the next-generation technologies such as sixth-generation (6 G) has further to be studied from the engineering and biomedical viewpoints. 

## Data Availability

Not applicable.
